# Antioxidant, Scavenging, Reducing, and Anti-Proliferative Activities of Selected Tropical Brown Seaweeds Confirm the Nutraceutical Potential of *Spatoglossum asperum*

**DOI:** 10.3390/foods10102482

**Published:** 2021-10-17

**Authors:** Bhakti Tanna, Babita Choudhary, Avinash Mishra, O. P. Chauhan, Manish Kumar Patel, Shadi Shokralla, Tarek K. Zin El-Abedin, Hosam O. Elansary, Eman A. Mahmoud

**Affiliations:** 1 Division of Applied Phycology and Biotechnology, CSIR—Central Salt and Marine Chemicals Research Institute, G. B. Marg, Bhavnagar 364002, India; tanna.bhakti90@gmail.com (B.T.); choudharybabita212@gmail.com (B.C.); patelm1402@gmail.com (M.K.P.); 2 Academy of Scientific and Innovative Research (AcSIR), CSIR, Ghaziabad 201002, India; 3 DRDO—Defence Food Research Laboratory, Siddarthanagar, Mysuru 570011, India; opchauhan@gmail.com; 4 Centre for Biodiversity Genomics, University of Guelph, Guelph, ON N1G 2W1, Canada; sshokral@uoguelph.ca; 5 Department of Integrative Biology, University of Guelph, Guelph, ON N1G 2W1, Canada; 6 Department of Agriculture & Biosystems Engineering, Faculty of Agriculture (El-Shatby), Alexandria University, Alexandria 21545, Egypt; drtkz60@gmail.com; 7 Plant Production Department, College of Food & Agriculture Sciences, King Saud University, Riyadh 11451, Saudi Arabia; 8 Department of Food Industries, Faculty of Agriculture, Damietta University, Damietta 34511, Egypt; emanmail2005@yahoo.com

**Keywords:** antioxidant, brown seaweeds, functional food, metabolites, metabolomics, nutraceuticals

## Abstract

Brown seaweeds have shown high potential of bioactivity and provide health benefits as an important functional food ingredient. Therefore, four abundantly growing tropical brown seaweeds—*Iyengaria stellata, Spatoglossum asperum*, *Sargassum linearifolium*, and *Stoechospermum polypodioides*—were collected from the Saurashtra Coast of the Arabian Sea. They were analyzed for metabolite profiling, biochemical activities (including total antioxidant, reducing, scavenging, and anti-proliferative characteristics), and total phenolic and flavonoid contents. A concentration-dependent antioxidant, reducing, and scavenging activities were observed for all four brown seaweeds. The *S. asperum* and *I. stellata* extracts showed maximum total antioxidant activity. *S. asperum* also showed high scavenging and reducing activities compared to other studied brown seaweeds. Further, *S. asperum* contained high total phenolic and flavonoid content compared to other brown seaweeds collected from the same coast. A multivariate correlation study confirmed a positive correlation between total phenolic and flavonoid contents, and biochemical activities (total antioxidant, scavenging and reducing) for all brown seaweeds. About 35% anti-proliferative activity was observed with *S. asperum* extract on Huh7 cells; in contrast *S. polypodioide* showed about 44% proliferation inhibition of Huh7 cells. Similarly, 26% proliferation inhibition of HeLa cells was observed with *S. asperum* extract. Overall, *S. asperum* possesses high total flavonoid and phenolic amounts, and showed potential antioxidant, scavenging and reducing characteristics. The study confirmed the nutraceutical potential of *S. asperum* and that it could be a promising functional food ingredient.

## 1. Introduction

The ocean is an extensive biome, in which a variety of life forms, starting from single-celled organisms to different types of multicellular organisms, develop, proliferate and die each day. It is presumed that the first life on Earth developed in the ocean. Additionally, it can be stated that single-cell algae eventually evolved to form each lifeform present today [1]. The macroscopic, multicellular algae found in enormous amount on rocky shores during low tides are called as seaweeds. Approximately 9000 seaweed species have been estimated to be available in different sizes, shapes and colors. Some of the seaweed species can form true aquatic forests, known as kelp forests, by reaching sizes of more than 50 m in area. Seaweeds are divided in three groups based on their pigmentation patterns: Phaeophyceae (brown), Rhodophyta (red), and Chlorophyta (green). However, other than visible color difference, there are also other factors responsible for their classification, such as the composition of the cell wall, photosynthetic pigments, the presence or absence of flagella and storage compounds [2]. Brown seaweeds are the second most profuse seaweeds, with almost 2000 species, which are mostly found to achieve their maximum biomass in temperate regions on rocky shores [3].

Due to the harsh environmental conditions of the oceans, seaweeds are exposed to different changes in climate, such as variations in temperature, mineral concentration, ultraviolet light exposure, and the concentration of dissolved gases, as well as attacks by pathogens and predators [4]. Due to these variations, seaweeds produce large amounts of different types of secondary metabolites that help them to survive in these changing environments and to perform important functions. These secondary metabolites are produced from the metabolism of primary metabolites, and are not directly involved in the reproduction, key-metabolism or survival of seaweeds. These secondary metabolites are responsible for a variety of bioactivities of seaweeds, such as antioxidant, antiviral, antifungal, anti-inflammatory, antimicrobial, anticoagulant and many other activities [5,6,7,8,9,10,11]. Polyphenolic compounds, including flavonoids, contain powerful antioxidant activity through various mechanisms, such as scavenging oxygen radicals, inhibiting oxidative enzymes or promoting anti-oxidases [12,13].

Researchers have been paying attention to the evolution of novel biologically active compounds from natural sources because nature has proved many times that it is an important resource of the compounds, which have biomedical properties [14,15,16]. Growing awareness of healthy food has increased interest in brown seaweeds due to their structurally diverse biological compounds, which play an important role in improving drugs and techno-functional foods [5,17]. Brown seaweeds are a rich source of variety of bioactive compounds, such as polyphenols, proteins and amino acids, polysaccharides, polyunsaturated fatty acids, minerals, fibers, vitamins, carotinoids and natural antioxidant compounds, which worldwide demand for them as a foodstuff [18]. Terrestrial plants have been overexploited as a resource of human nutrients, medical supplements and cosmetics; now brown seaweeds are being considered as an alternative resource for all these human requirements due to their rapid growing rate and large aquaculture size [19]. The end products or intermediates of metabolic pathways are classified as primary and secondary metabolites, in which primary metabolites are important for life, whereas secondary metabolites are essential for natural defense [20]. Brown seaweeds produce a specific group of polyphenols, known as phlolorotanins, as a secondary metabolites, which are synthesized during acetate malonate pathway. Phlorotanins play a vital role in the physiological processes of algae, as well as which they also play an important role in a number of significant mechanisms, including defense against the oxidative damage that occurs in response to various abiotic environmental stresses [21]. These phenolic compounds have become an interesting topic for researchers due to their broad health benefits and potential biological properties, such as anticancer, antioxidant, antiviral, antimicrobial, antidiabitic and anti-inflammatory activities. The research on algal phenolic compounds, nutritional and health benefits is mainly focused on their effective role in functional foods, pharmaceutical and nutraceutical applications, and cosmetics [22,23,24]. Cancer (uncontrolled cell proliferation) is a serious disease which is the leading cause of mortality in Europe and the USA, where it accounts for 20% and 14% of all deaths, respectively [25]. Studies revealed that reason behind uncontrolled cell proliferation (cancer) is mostly due to unhealthy dietary intake and lifestyle, and still it is a challenge for the medical profession to prevent and treat cancer. A large number of epidemiological studies have revealed that seaweed consumption in association with a healthier diet profile lowers the incidence of various chronic diseases, including cardiovascular diseases and cancer [5,26]. The secondary metabolites isolated from seaweeds showed anti-proliferative activities through various mechanisms, such as inhibition of cancer cell growth and invasion, by inducing apoptosis in cancer cells, regulating cancer-linked genes and metastasis [18,27,28].

Metabolomics is a new approach in the ‘omics’ techniques, and it represents the metabolite activities in an organism at a particular time. Seaweeds are known to possess a variety of metabolic activities, and the study of their metabolomics might unfold different aspects of seaweeds’ properties, and also identify their nutraceutical application [28]. Brown seaweeds have been reported to have nutraceutical and biomedical potential in cancer therapy [8,10], as they show anti-proliferative activities [5]. The present study was undertaken with the objective to determine the different biological activities of the brown seaweeds. Four abundantly growing brown seaweeds (*Iyengaria stellata, Spatoglossum asperum*, *Sargassum linearifolium*, *Stoechospermum polypodioides*) were harvested from the Arabian sea (Saurashtra Coast, Gujarat), India to analyze the total flavonoid and phenolic contents, scavenging, total antioxidant and reducing activities, and anti-proliferative activity on human cancer cell lines (HeLa and Huh7). The results confirmed the nutraceutical potential of the brown seaweeds [29,30,31,32].

## 2. Materials and Methods

### 2.1. Collection of Seaweed Samples

Brown seaweeds, namely *Iyengaria stellata* (Børgesen) Børgesen (IS), *Sargassum linearifolium* (Turner) C.Agardh (SL), *Spatoglossum asperum* J.Agardh (SA)*,* and *Stoechospermum polypodioides* (J.V.Lamouroux) J.Agardh (SP) grow abundantly in the Saurashtra coast of the Arabian Sea. These four brown seaweeds were collected from the Veraval (20°53′8.93″ N; 70°22′5.05″ E), Okha (22°28′8.19″ N; 69°04′8.24″ E), and Adri (20°57′.42″ N; 70°16′.32″ E) coasts [13]. Collected samples were thoroughly washed with seawater, freeze-dried and stored at −80 °C for further use. Simultaneously, another set of washed samples were shed-dried for approximately 3–4 days until constant weight was achieved, and stored for further analysis.

### 2.2. Metabolite Extraction and Analysis

Metabolites were isolated and quantified using optimized method [33,34,35]. Briefly, dried-powered seaweed samples (100 mg) were extracted with cold methanol by vortexing, incubating at 70 °C for 10 min, and centrifugation at 11,000× *g* at 4 °C for 10 min. Supernatant was collected, again extracted with chloroform and, upper-phase was aspirated, dried and derivatized. For the quantification of metabolites, ribitol/adonitol (6 μg) was added as an internal reference. Methoxyamine hydrochloride (40 µL) and *N*-Methyl-*N*-(trimethylsilyl) trifluoroacetamide (70 µL) were used for the derivatization. The derivatized samples were analyzed by gas chromatography and mass spectroscopy (GC-MS, Shimadzu, Japan). Mass spectra peaks were recorded and compared with the NIST library for the identification. The quantification was performed using internal standard.

### 2.3. Preparation of Seaweed Extract

About 10 g of dry seaweed samples (passed through sieve) was used for the extraction with aqueous methanol (70%, *v*/*v*, 500 mL) on magnetic stirrer for overnight. The supernatant was collected after centrifugation of extraction mixture at 7000× *g* for 10 min. The extraction procedure repeated twice. All three supernatants were pooled and concentrated under 100–150 mbar at 37 °C using rotary evaporator and then lyophilized. The lyophilized extracts were stored at −20 °C for further use.

### 2.4. Total Antioxidant Activity

Total antioxidant activity was performed by estimating scavenging of ABTS free radicals, which were generated by mixing potassium persulphate (2.45 mM) with ABTS diammonium salt (7 mM), followed by 12–16 h incubation in dark room at room-temperature, and trolox was used as standard [36,37,38]. The stock solution of ABTS free radicals was diluted with water until the working solution was prepared, which had the absorbance of 0.70 ± 0.02 at 734 nm. Various (200–1000 μg·mL^−1^) concentrations of seaweed extracts or trolox (standard) was mixed with ABTS free radicals (1 mL), and was incubated for 90 min. The absorbance was read at 734 nm and the activity of samples was compared with standards and percentage inhibition was calculated [29].

### 2.5. Radical Scavenging Assay

DPPH (2,2-diphenyl-1-picrylhydrazyl) is a free radical. Evaluation of DPPH scavenging activity was carried out by preparing DPPH stock solution (0.024%, *w*/*v*, in methanol) and diluting it with methanol until 0.98 ± 0.02 absorbance was obtained at 517 nm [39]. A measure of 1 mL of working solution was mixed with various (200–1000 μg·mL^−1^) concentrations of seaweed extracts or trolox (standard) and was incubated overnight at room temperature in the dark. Sample absorbance was taken at 517 nm, which was compared with standard curve to obtain final scavenging activity of the extract [40,41].

### 2.6. Total Flavonoid and Phenolic Contents

Total flavonoid (TFC) and phenolic (TPC) contents were determined with the help of standards (quercetin and gallic acid [13,38,42]. For TPC, different concentrations of seaweed extracts (200–1000 μg·mL^−1^) were mixed with 2.5 mL of Folin–Coicalteu (FC) reagents (0.2 M) (Sigma, St. Louis, MI, USA), incubated for 5 min and sodium carbonate (2 mL; Na_2_CO_3_; 75 g L^−1^) was added, which was further incubated (90 min) at room temperature in dark condition. Afterwards, absorbance of the reaction mixture was taken at 760 nm, and TPC of extracts was calculated as mg·mL^−1^ gallic acid per 100 mg of extract. For TFC, different concentrations of seaweed extracts (200–1000 μg·mL^−1^) were mixed with 0.3 mL NaNO_2_ (5%, *v*/*v*), incubated at room temperature for 5 min and 0.3 mL AlCl_3_ (10%, *v*/*v*) and 2 mL NaOH (1 M) were added to the reaction mixture. Absorbance was read at 510 nm, and TFC was calculated using quercetin standard curve as mg·mL^−1^ quercetin per 100 mg of extract 13 [13,38,42].

### 2.7. Reducing Power

The reducing power of seaweed extracts was calculated by using ascorbic acid as standard. Different concentrations of seaweeds extracts (100–500 μg·mL^−1^) were mixed with 1 mL of phosphate buffer (0.2 M, pH 6.6) and 1 mL of K_3_Fe(CN)_6_ (10 mg·mL^−1^) and was then incubated for 20 min at 50 °C in water bath (Julabo, Seelbach, Germany). The reaction was ended by addition of 1 mL of TCA (100 mg L^−1^) in the reaction mixture. Supernatant was collected after centrifugation at 7000× *g* for 10 min at room temperature. Freshly prepared FeCl_3_ (0.1%, *w*/*v*) was mixed with collected supernatant and incubated for 10 min at room temperature. Absorbance was taken at 700 nm to calculate reducing power [34,43].

### 2.8. Cell Culture and Anti-Proliferative Activity

The human cervical cancer cell line (HeLa) and human hepatoma cancer cell line (Huh7), purchased from National Centre for Cell Science (NCCS), Pune (India) were maintained in MEM (2 mM L-glutamine, 1 mM sodium pyruvate, NEAA, 1.5 g L^−1^ sodium bicarbonate) and DMEM:F12 (1:1 Mixture; 2 mM L-glutamine, 15 mM HEPES buffer, 1.5 g L^−1^ sodium bicarbonate and trace elements) medium, respectively at 37 °C under 5% CO_2_ in the air. Both media were supplemented with heat inactivated Fetal Bovine Serum and antibiotic antimycotic solution (with 10,000 unit’s penicillin, 10 mg streptomycin and 25 µg amphotericin B per ml in 0.9% normal saline). The cells were used only up to 20 passages for all the experiments. MTT was used to measure anti-proliferative activity [44,45]. Briefly, 10,000 cells were seeded in each well with their respective media and incubated for 24 h at 37 °C under 5% CO_2_. Seaweed extracts were added to 96 well-plate, followed by addition of PBS and at the final concentration of 10% and incubation for 2.5 h. Absorbance was taken at 570 nm and anti-proliferative activity was calculated using following equation and compared with control (cells growing without extract).

(1) $$\mathrm{Anti}-\mathrm{proliferative}\;\mathrm{activity}\;\left(\%\right)=100\times \left[1-\frac{\left({\mathrm{abs}}_{570\left(\mathrm{sample}\right)}-{\mathrm{abs}}_{690\left(\mathrm{blank}\right)}\right)}{\left({\mathrm{abs}}_{570\left(\mathrm{control}\right)}-{\mathrm{abs}}_{690\left(\mathrm{blank}\right)}\right)}\right]$$

### 2.9. Statistical Analysis

Data were shown as average (mean) ± SE (standard error of the mean). ANOVA (Analysis of variance) and Tukey’s honestly significant difference (HSD) were applied for statistical analysis and statistical differences were expressed with different letters at significance *p* < 0.05. Correlation and multivariate analyses were performed for biochemical activities, TPC, and TFC, using Pearson’s correlation matrix and PCA (principal component analysis).

## 3. Results

### 3.1. Total Antioxidant, Scavenging, and Reducing Activities

**Figure 1:**
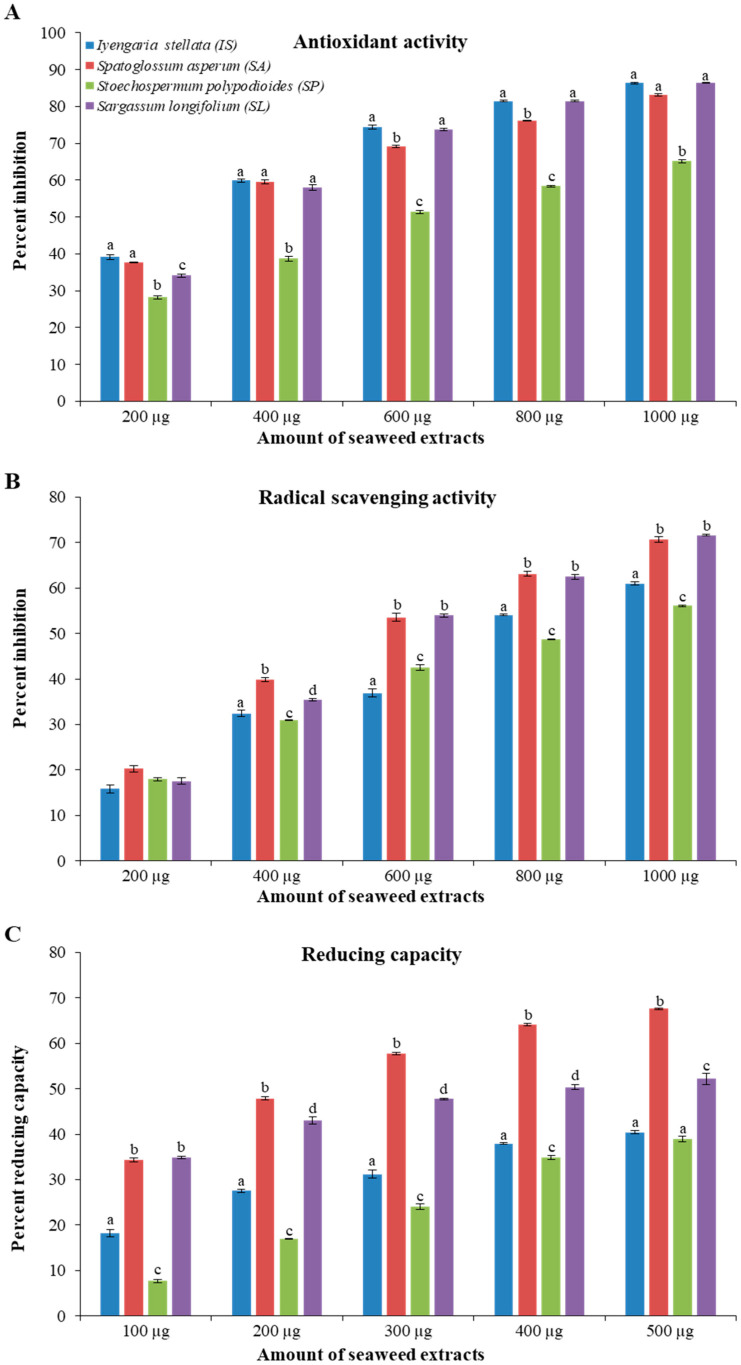
Biochemical activities of selected brown seaweeds. (**A**) Total antioxidant activity, (**B**) scavenging activity, and (**C**) reducing activity. All activities are shown as mean ± SE (*n* = 3) and different small letters (e.g., a, b, c, d) indicate a statistically significant difference (Tukey test *p* < 0.05).

All seaweed extracts showed concentration-dependent antioxidant, scavenging, and reducing activities (Figure 1). The seaweeds IS and SA showed about 60% antioxidant activity with 400 μg extract-concentration, followed by SL (57%) and SP (51%) with the concentration doses of 400 and 600 μg, respectively. About 86% of ABTS free radical inhibition was observed, with the highest concentration of 1000 μg for IS and SL, whereas 83% and 65% inhibition were observed for SA and SP, respectively, with the same extract concentration (Figure 1A).

The highest scavenging activity was observed with SA and SL, which was 53% with 600 μg extract-dose, while at a dose of 1000 μg, both showed 70% inhibition. With the 1000 μg dose, IS and SP showed only 60% and 56% scavenging activity, respectively (Figure 1B). SA also showed the highest reducing activity (67%) with 1000 μg concentration followed by SL (52%), IS (40%) and SP (39%) with 1000 μg extract-dose (Figure 1C).

**Figure 2:**
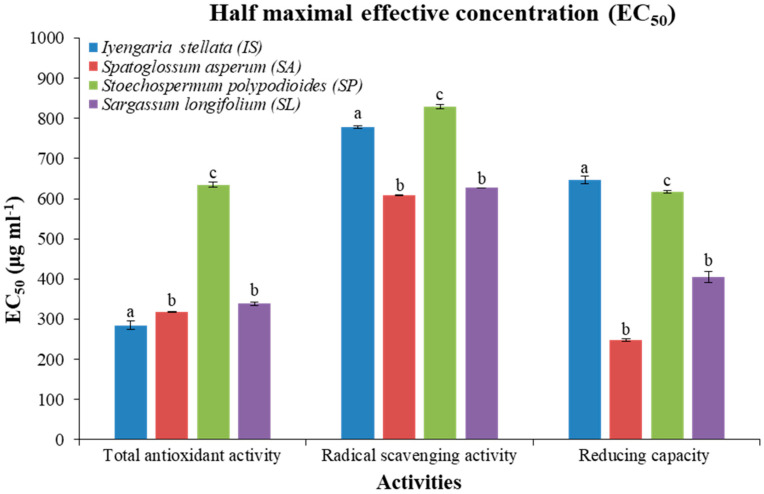
EC_50_ (half maximal effective concentration; μg·mL^−1^) of brown seaweeds for different biochemical activities. Data are shown as mean (μg·mL^−1^) ± SE (*n* = 3) and different small letters (e.g., a, b, c) indicate a statistically significant difference (Tukey test *p* < 0.05).

Seaweed IS showed about 285 ± 10 μg·mL^−1^ half maximal effective concentration (EC_50_) for antioxidant activity followed by SA (320 ± 2 μg·mL^−1^), SL (340 ± 5 μg·mL^−1^) and SP (635 ± 5 μg·mL^−1^). Furthermore, SA showed about 610 ± 2 and 250 ± 3 μg·mL^−1^ EC_50_ dose for scavenging and reducing activities, respectively. Overall, SA showed the lowest EC_50_ dose (except for antioxidants, which were non-significant for IS), therefore showing maximum activities (Figure 2).

### 3.2. Total Flavonoid and Phenolic Content

**Figure 3:**
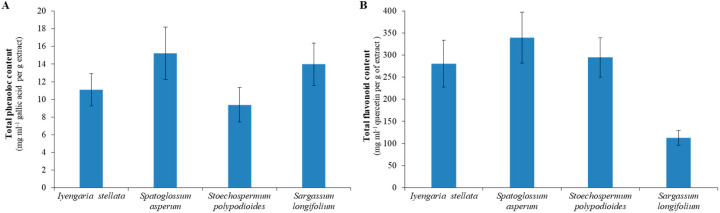
Total phenolic and flavonoid contents of selected brown seaweeds. (**A**) TPC (total phenolic content) is shown as GAE (mg·mL^−1^) per gram of extract. (**B**) TFC (total flavonoid amount) is shown as quercetin equivalent (mg·mL^−1^) per gram of extract. All data are mean ± SE (*n* = 3).

High TPC was estimated with SA (15 ± 3 mg·mL^−1^·g extract) followed by SL (14 ± 2 mg·mL^−1^·g extract), IS (11 ± 2 mg·mL^−1^·g extract) and SP (9 ± 2 mg·mL^−1^·g extract) (Figure 3A). Similarly, the maximum TFC was found in SA (340 ± 60 mg·mL^−1^·g extract), followed by SP (295 ± 45 mg·mL^−1^·g extract), and IS (280 ± 50 mg·mL^−1^·g extract), whereas the lowest TFC (113 ± 17 mg·mL^−1^ quercetin per g extract) was observed in SL (Figure 3B).

### 3.3. Correlation Analysis

A very strong (0.9–1.0) and strong correlations (0.7–0.9) were observed between TPC and biochemical characteristics (scavenging, total antioxidant and reducing) for SP and SL seaweed extracts, respectively (Figure S1 and Table S1). In contrast, moderate (0.4–0.7) correlation was found between TPC and biochemical activities for IS and SA seaweeds. Similarly, there was a very strong and strong correlation between TFC and biochemical activities for SP and SL seaweeds, respectively (Figure S2 and Table S1). Among biochemical characteristics, scavenging, total antioxidant and reducing activities showed very strong correlation (0.9–1.0) to each other for all seaweeds. Interestingly, a strong correlation was found between TPC and TFC for the IS, SP and SL seaweeds; however, SA showed a weak (0.1–0.4) correlation.

### 3.4. Anti-Proliferative Activity

**Figure 4:**
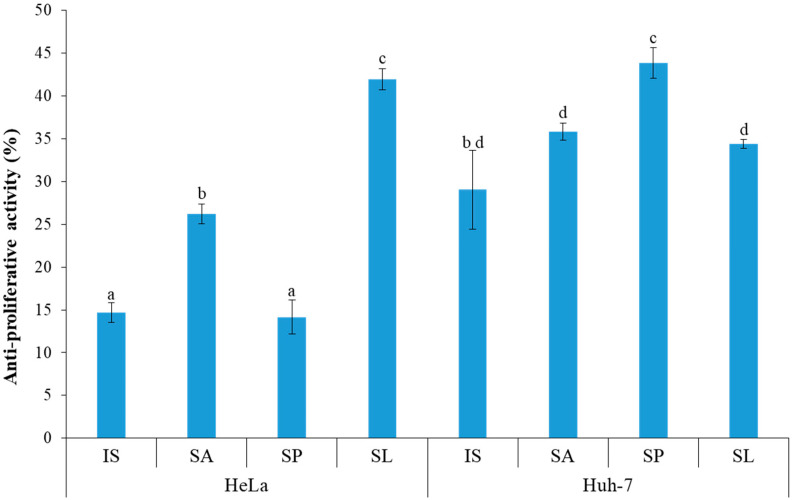
Anti-proliferative property of selected brown seaweed extracts on carcinoma cell lines. Extracts were demonstrated on the propagation HeLa and Huh7 using MTT assay. All values are expressed as mean ± standard error of the mean (SE; *n* = 3). Values are expressed as mean ± standard error of the mean (SE; *n* = 4) and different small letters (e.g., a, b, c, d) indicate a statistically significant difference (Tukey test *p* < 0.05).

The SP and SL seaweed extracts showed high inhibition of cell proliferation, approximately 44% and 41% for Huh7 and HeLa cells, respectively, compared to their controls, i.e., untreated cells (cells growing in their respective media without any addition of seaweed extract) (Figure 4). About 35% inhibition was found with SA and SL extracts on Huh7 cells growth. In contrast, the IS and SP extracts inhibited HeLa propagation only by about 14%. About 26% and 29% anti-proliferation activity was observed with SA and IS on HeLa and Huh7 cells, respectively.

### 3.5. Metabolite Profiling and Quantification

A total of 68 different types of metabolites were quantified in the brown seaweeds (Table S2). These metabolites belonged to different groups including sugars, sugar-derivatives, amino-acids, fatty acids, organic compounds and polyol compounds. The maximum 25 different metabolites were identified in IS and SP, followed by 21 in SA and 19 in SL. Notably, IS was found to be rich in sugar alcohol, glucitol (7474 ± 886 µg·g^−1^ DW), followed by meso-erythritol (453 ± 46 µg·g^−1^ DW). Similarly, a commercially important low-calorie sugar, psicose (76 ± 17 µg·g^−1^ DW) was estimated in SP. A high sucrose level (1097 ± 76 µg·g^−1^ DW) was estimated in SL, followed by SP (506 ± 37 µg g^−1^ DW). A number of amino-acids were estimated in IS compared to the other three brown seaweeds. IS has the highest concentration of metabolites (about 10 mg·g^−1^) followed by SL (2 mg·g^−1^), SP (1.7 mg·g^−1^) and SA (1 mg·g^−1^).

### 3.6. Multivariate Correlation Analysis

**Figure 5:**
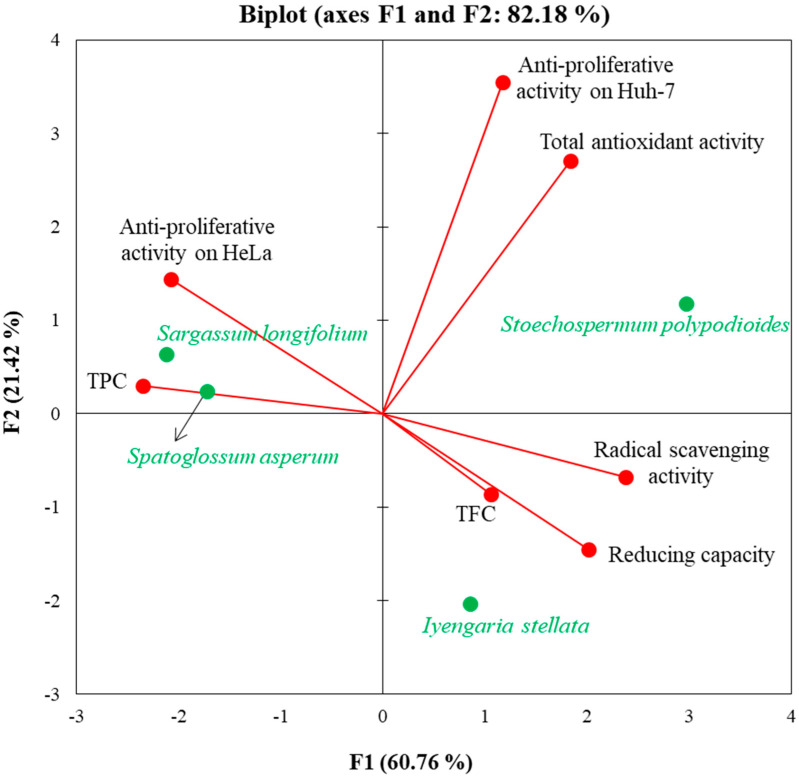
Principal component analysis. A Biplot of different bioactivities of brown seaweeds inferred from the Pearson correlation matrix.

A cumulative correlation and principal component analysis (PCA) were conducted for the selected tropical brown seaweeds in a combination of biochemical (scavenging, total antioxidant, and reducing) activities, contents (TFC and TPC), and anti-proliferative activity (Figure 5 and Table S3). Overall, antioxidant activity showed a weak (0.1–0.4 coefficient) correlation with scavenging (R^2^ = 0.395), reducing (R^2^ = 0.161) and anti-proliferative activity of HeLa cells (R^2^ = 0.158). However, a strong correlation was found with the proliferation inhibition of Huh7 cells (R^2^ = 0.853). Further, the antioxidant activity did not show any correlation (0.0–0.1 coefficient) with TFC (R^2^ = 0.027); however, a moderate correlation with TPC (R^2^ = 0.466) was found. A very strong correlation (R^2^ = 0.978) was noticed between scavenging activity and TPC; however, a strong correlation was found for reducing activity with scavenging activity (R^2^ = 0.865) and TPC (R^2^ = 0.877). Further, there was no correlation of TFC with antioxidant (R^2^ = 0.027), reducing (R^2^ = 0.002) activity, TPC (R^2^ = 0.038) and proliferation inhibition of Huh7 cells (R^2^ = 0.0.29); however, it was moderately correlated with inhibition of HeLa cells (R^2^ = 0.623), and showed weak correlation with scavenging activity (R^2^ = 0.102). TPC showed a moderate correlation with the anti-proliferative activity of HeLa cells (R^2^ = 0.537), while showing a weak correlation with the anti-proliferative activity of Huh7 cells (R^2^ = 0.125). All activities (scavenging, total antioxidant, and reducing) showed a proportional correlation (Pearson correlation matrix with positive sign) to each other, and also with TFC and anti-proliferation of Huh7 cells. Interestingly, TPC showed an inversely proportional correlation (Pearson correlation matrix with –ve sign) with all activities except for the anti-proliferation of HeLa cells (positive; 0.733). The proliferation inhibition of HeLa cells was inversely proportionally correlated with all activities (scavenging, total antioxidant, and reducing), TFC and TPC (Table S3). No correlation (R^2^ = 0.038) was observed between TPC and TFC.

The PCA showed a statistical difference among different bioactivities, contents (TFC and TPC), and proliferative inhibition activities for the tropical brown seaweeds (Figure 5). The biplot deduced from PCA supported approximately 82.18% variation and sorted the brown seaweeds by their nutraceutical potential. The PCA showed that SP has high total antioxidant activity and also inhibits the proliferation of Huh7 cells. Similarly, SA is rich source of total flavonoid contents, whereas SL possesses anti-proliferation potential of HeLa cells. Furthermore, IS had high total phenolic contents, and also contributed more to scavenging and reducing activities (Figure 5) than was found in prior studies on tropical brown seaweeds.

## 4. Discussion

Seaweeds have been used as food from ancient times in Asian countries, such as China, Japan and Korea; however, they were only used for industrial purposes in Western countries. Algae and their extracted constituents were included as novel foods and novel food ingredients in January 1997 as CE 258/97 regulation of the European Parliament and of the Council. The food industry has been allowed to use seaweeds in their raw or processed form as ingredients in their formulations or as additives. Hence, there has been an increase in awareness regarding the use of seaweeds and their components in pharmaceuticals, cosmetics, nutraceuticals and biotechnological applications [46].

In the present study, all seaweed extracts showed dose-dependent biochemical activities (Figure 1), and the lowest dose of EC_50_ was observed with extract from *Iyengaria stellata*, followed by an extract from *Spatoglossum asperum* for total antioxidant activity. Similarly, the extracts obtained from *S. asperum* showed the lowest dose of EC_50_ for scavenging and reducing activities (Figure 2). The methanolic extracts from the brown seaweeds harvested from India also showed dose-dependent total antioxidant activity [47]. Methanol extracts of *S. asperum* were highlighted for their high potential total antioxidant, scavenging and reducing activities compared to their respective standards [48].

High total phenolic content and total flavonoid were observed in the brown seaweed *S. asperum* (Figure 3) compared to other abundantly grown brown seaweeds. Phenolic compounds and flavonoids are well known for their high bioactivities, including total antioxidants, scavenging and reducing [13]. A very strong correlation was observed in the study between TPC and biochemical activities for each seaweeds (Figure S1 and Table S1). Similarly, TFC also showed a strong correlation with biochemical activities of all studied brown seaweeds (Figure S2 and Table S1). Phaeophyceae (brown seaweeds) are known for their high amounts of phenolic compounds. Polyphenolic compounds are useful for their anti-ageing, anticancer, antibacterial and many other beneficial health effects [49]. Júnior et al. also correlated the high antioxidant activity of the brown seaweed *Spatoglossum* to the presence of flavonoids [48]. Earlier studies also demonstrated the synergy in biochemical activities, and phenolic and flavonoid content in brown seaweeds [50,51]. Some metabolites identified by GC-MS in the brown seaweeds (Table S2) may also play a role in their biochemical activities. In land plants, such as *Suaeda* species, differential accumulation of certain metabolites, such as amino acids, sugars, and organic acids, was shown under varying stress conditions [52]. The accumulation of metabolites was linked with various biological activities, such as scavenging, total antioxidants, reducing and anti-proliferative activities. The metabolites determined in seaweeds play a major role in the different metabolic pathways [5,34]. The high content of sugar derivatives, including sugar alcohol, commonly known as polyol, was determined in the brown seaweeds. Polyol compounds play a very important role in glycolysis, lipid metabolism, glycogen heterogenesis and many other pathways. Most of them are involved in galactose metabolism, and galactose can be converted from sorbitose, glycerol, mannose and galactositol by the enzyme activities. Sugars including galactose, fructose, and maltose also play a crucial role in the metabolism of sucrose/starch. Sucrose itself is an extracellular material that participates in the metabolism of starch and sucrose. Mannitol also shows its role in the metabolism of mannose and fructose, which further are converted into fructose by enzymes. Some amino acids and fatty acids were also observed in the brown seaweeds. Amino acids are directly involved in many metabolic pathways, such as glutamic acid and glycine, which are regulators for the glutathione metabolism. Fatty acids provide membrane rigidity and thus protect seaweeds from harsh environments.

Phenolic and flavonoid compounds from marine algae are well known for their anti-proliferative activities [3,53,54,55]. Extracts obtained from *S. asperum* showed 35% anti-proliferation of Huh7 cells and 26% of HeLa cells (Figure 4). Selected brown seaweeds from the Mandapam Coast of Tamil Nadu (India) showed resazurin-based growth inhibition of A549, HCT-15, MG-63 and PC-3 cell lines [45]. They correlated with the high growth inhibition observed with higher values of total flavonoid content in seaweed. In this study, TPC and TFC showed a positive correlation of anti-proliferative activity with HeLa cells and Huh7 cells, respectively (Table S3). PCA confirmed that *S. asperum* contained high TPC content and, therefore, high anti-proliferation of HeLa cells (Figure 5). The edible brown seaweed *S. asperum* is considered a potential source of natural antioxidant molecules and different bioactive metabolites [47,56,57].

## 5. Conclusions

In conclusion, *S. asperum* showed potential antioxidant, scavenging and reducing activities among studied, tropical, abundantly grown brown seaweeds from the same coast. The study confirms the nutraceutical potential of the brown seaweed, *S. asperum*, and also recommends brown seaweeds to be a part of daily diet because of their beneficial health properties.

## Data Availability

Not applicable.

## Supplementary Materials

The following are available online at https://www.mdpi.com/article/10.3390/foods10102482/s1, Figure S1: Correlation between different activities (total antioxidant, scavenging and reducing) and total phenolic contents (TPC) of the selected brown seaweeds. Dotted lines represent linear regression curves. Figure S2: Correlation between different activities (total antioxidant, scavenging and reducing) and total flavonoid contents (TFC) of the selected brown seaweeds. Dotted lines represent linear regression curves. Table S1: Correlation matrix of different activities (total antioxidant, scavenging and reducing) and phenolic (TPC) and flavonoid (TFC) contents of the selected brown seaweeds, Table S2: Metabolites identified from abundantly grown brown seaweeds by gas, Table S3: Correlation matrix plots for PCA analysis of different activities (total antioxidant, scavenging, reducing and anti-proliferative) and contents (TPC and TFC) of the selected brown seaweeds.
