# Supplementary material for: Antioxidant, Scavenging, Reducing, and Anti-Proliferative Activities of Selected Tropical Brown Seaweeds Confirm the Nutraceutical Potential of Spatoglossum asperum
Source: Foods. 2021 Oct 17;10(10):2482. doi: 10.3390/foods10102482 (PMC8536100; doi:10.3390/foods10102482)
Supplement: Supplementary file 1 [file foods-10-02482-s001.zip › foods-1399538-SI.pdf]

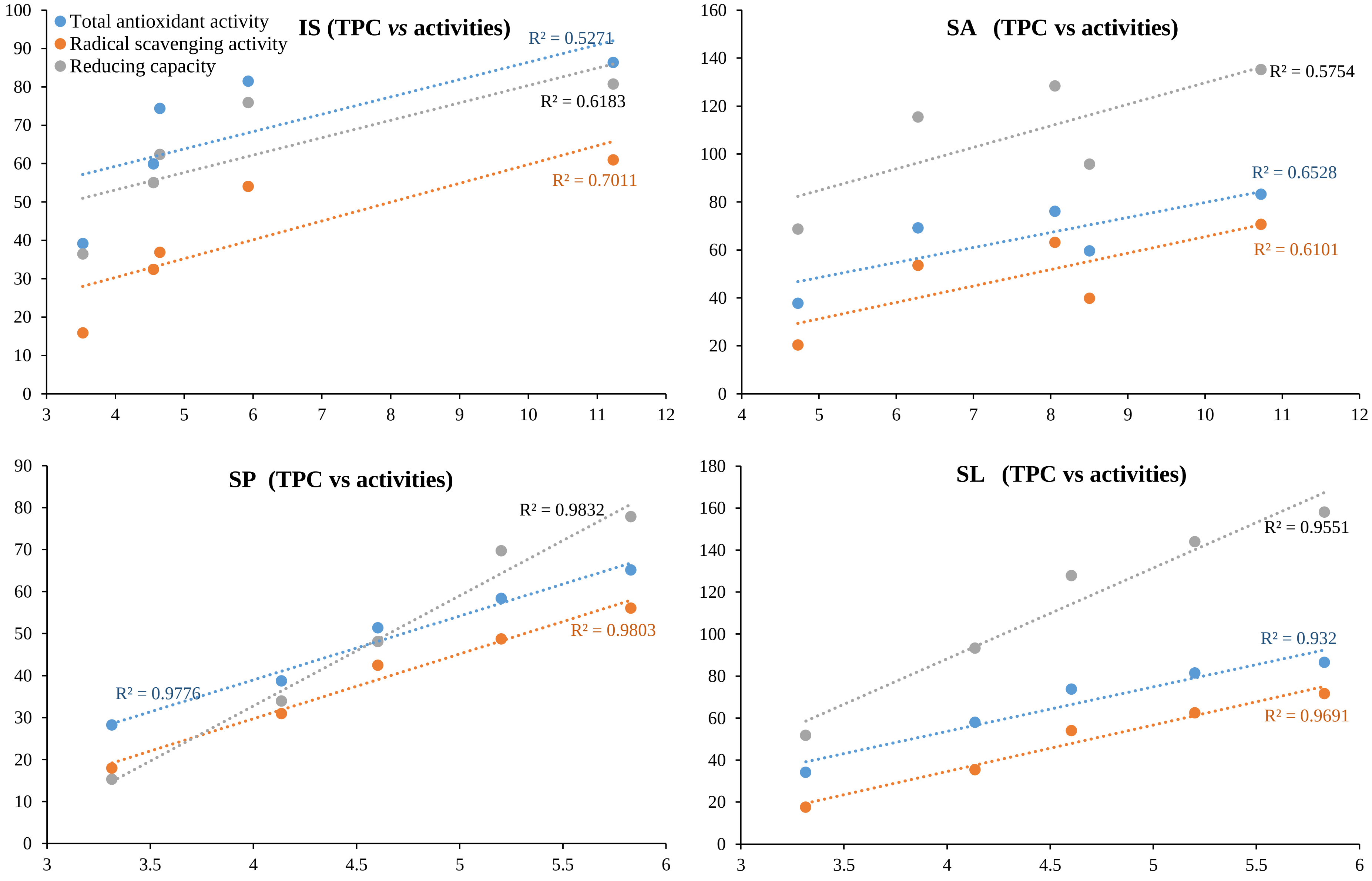

Figure S1: Correlation between different activities (total antioxidant, scavenging and reducing) and total phenolic contents (TPC) of the selected brown seaweeds. Dotted lines represent linear regression curves.

IS: *Iyengaria stellate*; SA: *Spatoglossum asperum*; SP: *Stoechospermum polypodioides*; SL: *Sargassum linearifolium*

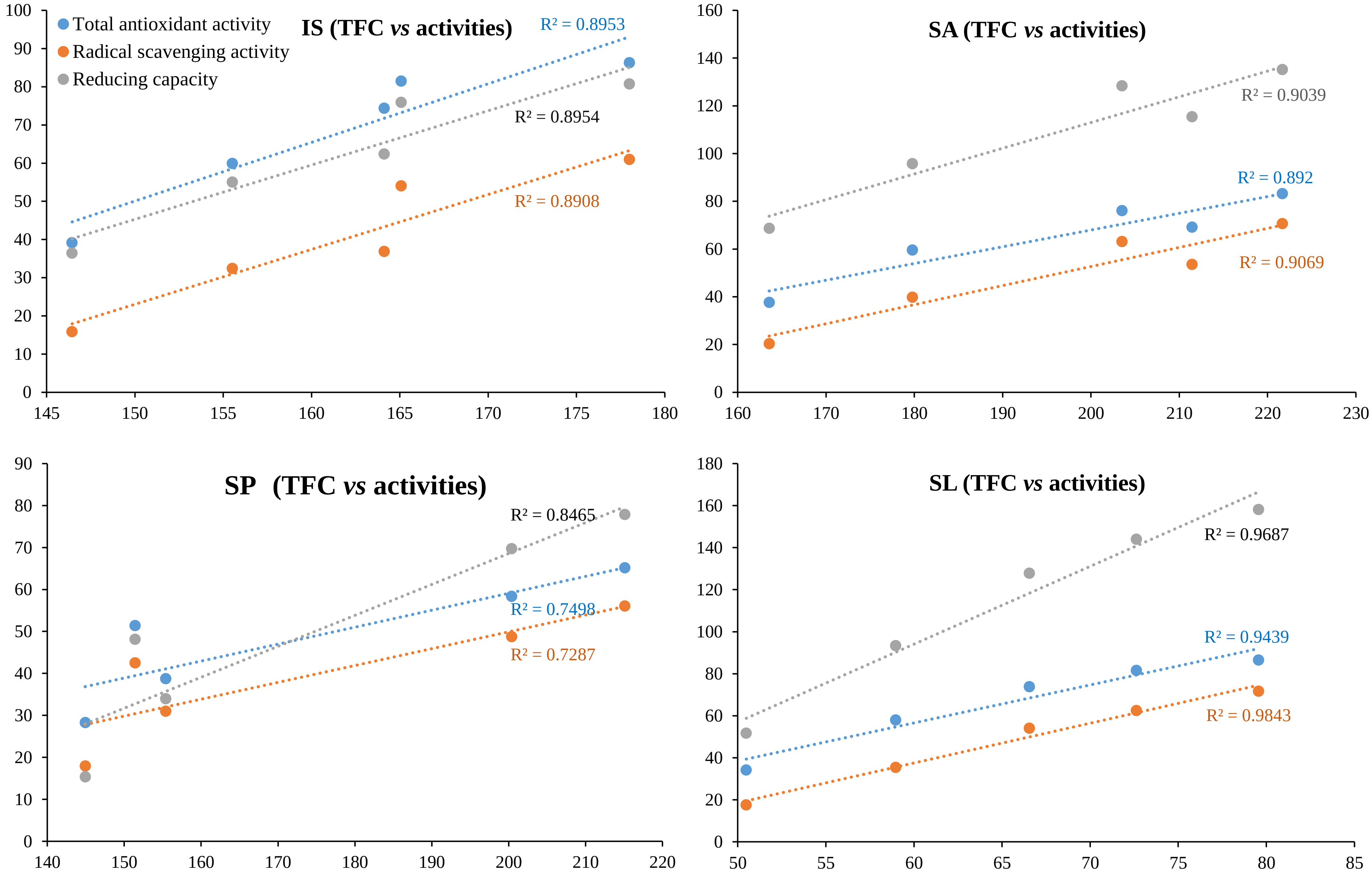

Figure S2: Correlation between different activities (total antioxidant, scavenging and reducing) and total flavonoid contents (TFC) of the selected brown seaweeds. Dotted lines represent linear regression curves.

IS: *Iyengaria stellate*; SA: *Spatoglossum asperum*; SP: *Stoechospermum polypodioides*; SL: *Sargassum linearifolium*

Table S1: Correlation matrix of different activities (total antioxidant, scavenging and reducing) and phenolic (TPC) and flavonoid (TFC) contents of the selected brown seaweeds.

Iyengaria stellata (Børgesen) Børgesen

Correlation matrix (Pearson):

Variables	TPC	ABTS	DPPH	RP	TFC
TPC	**1**	0.726	0.837	0.786	**0.892**
ABTS	0.726	**1**	**0.958**	**0.985**	**0.946**
DPPH	0.837	**0.958**	**1**	**0.992**	**0.944**
RP	0.786	**0.985**	**0.992**	**1**	**0.946**
TFC	**0.892**	**0.946**	**0.944**	**0.946**	**1**

Values in bold are different with a significance level alpha=0.05

p-values (Pearson):

Variables	TPC	ABTS	DPPH	RP	TFC
TPC	**0**	0.165	0.077	0.115	**0.042**
ABTS	0.165	**0**	**0.010**	**0.002**	**0.015**
DPPH	0.077	**0.010**	**0**	**0.001**	**0.016**
RP	0.115	**0.002**	**0.001**	**0**	**0.015**
TFC	**0.042**	**0.015**	**0.016**	**0.015**	**0**

Coefficients of determination (Pearson):

Variables	TPC	ABTS	DPPH	RP	TFC
TPC	**1**	0.527	0.701	0.618	0.796
ABTS	0.527	**1**	0.918	0.971	0.895
DPPH	0.701	0.918	**1**	0.985	0.891
RP	0.618	0.971	0.985	**1**	0.895
TFC	0.796	0.895	0.891	0.895	**1**

Spatoglossum asperum J.Agardh

Correlation matrix (Pearson):

Variables	TPC	ABTS	DPPH	RP	TFC
TPC	**1**	0.808	0.781	0.759	0.654
ABTS	0.808	**1**	**0.994**	**0.994**	**0.944**
DPPH	0.781	**0.994**	**1**	**0.999**	**0.952**
RP	0.759	**0.994**	**0.999**	**1**	**0.951**
TFC	0.654	**0.944**	**0.952**	**0.951**	**1**

Values in bold are different with a significance level alpha=0.05

p-values (Pearson):

Variables	TPC	ABTS	DPPH	RP	TFC
TPC	**0**	0.098	0.119	0.137	0.232
ABTS	0.098	**0**	**0.000**	**0.001**	**0.016**
DPPH	0.119	**0.000**	**0**	**< 0.0001**	**0.012**
RP	0.137	**0.001**	**< 0.0001**	**0**	**0.013**
TFC	0.232	**0.016**	**0.012**	**0.013**	**0**

Coefficients of determination (Pearson):

Variables	TPC	ABTS	DPPH	RP	TFC
TPC	**1**	0.653	0.610	0.575	0.427
ABTS	0.653	**1**	0.989	0.988	0.892
DPPH	0.610	0.989	**1**	0.997	0.907
RP	0.575	0.988	0.997	**1**	0.904
TFC	0.427	0.892	0.907	0.904	**1**

Stoechospermum polypodioides (J.V.Lamouroux) J.Agardh

Correlation matrix (Pearson):

Variables	TPC	ABTS	DPPH	RP	TFC
TPC	**1**	**0.989**	**0.990**	**0.992**	**0.909**
ABTS	**0.989**	**1**	**0.998**	**0.990**	0.866
DPPH	**0.990**	**0.998**	**1**	**0.987**	0.854
RP	**0.992**	**0.990**	**0.987**	**1**	**0.920**
TFC	**0.909**	0.866	0.854	**0.920**	**1**

Values in bold are different with a significance level alpha=0.05

p-values (Pearson):

Variables	TPC	ABTS	DPPH	RP	TFC
TPC	**0**	**0.001**	**0.001**	**0.001**	**0.032**
ABTS	**0.001**	**0**	**0.000**	**0.001**	0.058
DPPH	**0.001**	**0.000**	**0**	**0.002**	0.066
RP	**0.001**	**0.001**	**0.002**	**0**	**0.027**
TFC	**0.032**	0.058	0.066	**0.027**	**0**

Coefficients of determination (Pearson):

Variables	TPC	ABTS	DPPH	RP	TFC
TPC	**1**	0.978	0.980	0.983	0.827
ABTS	0.978	**1**	0.996	0.980	0.750
DPPH	0.980	0.996	**1**	0.974	0.729
RP	0.983	0.980	0.974	**1**	0.846
TFC	0.827	0.750	0.729	0.846	**1**

Sargassum longifolium (Turner) C.Agardh

Correlation matrix (Pearson):

Variables	TPC	ABTS	DPPH	RP	TFC
TPC	**1**	**0.965**	**0.984**	**0.977**	**0.998**
ABTS	**0.965**	**1**	**0.991**	**0.998**	**0.972**
DPPH	**0.984**	**0.991**	**1**	**0.998**	**0.992**
RP	**0.977**	**0.998**	**0.998**	**1**	**0.984**
TFC	**0.998**	**0.972**	**0.992**	**0.984**	**1**

Values in bold are different with a significance level alpha=0.05

p-values (Pearson):

Variables	TPC	ABTS	DPPH	RP	TFC
TPC	**0**	**0.008**	**0.002**	**0.004**	**0.000**
ABTS	**0.008**	**0**	**0.001**	**0.000**	**0.006**
DPPH	**0.002**	**0.001**	**0**	**0.000**	**0.001**
RP	**0.004**	**0.000**	**0.000**	**0**	**0.002**
TFC	**0.000**	**0.006**	**0.001**	**0.002**	**0**

Coefficients of determination (Pearson):

Variables	TPC	ABTS	DPPH	RP	TFC
TPC	**1**	0.932	0.969	0.955	0.995
ABTS	0.932	**1**	0.982	0.995	0.944
DPPH	0.969	0.982	**1**	0.995	0.984
RP	0.955	0.995	0.995	**1**	0.969
TFC	0.995	0.944	0.984	0.969	**1**

Table S2: Metabolites identified from abundantly grown brown seaweeds by gas chromatography-mass spectrometry (GC-MS) analysis.

Category	Metabolites	IS	SA	SP	SL
Sugar	2-Deoxyribose	nd	nd	18 ± 5	2 ± 0.6
	3-alpha.-Mannobiose	nd	nd	nd	5 ± 1
	4-Ketoglucose	nd	nd	37 ± 5	30 ± 1
	Erythrose	nd	8 ± 3	5 ± 2	2 ± 0.1
	Fructose	nd	Nd	155 ± 81	nd
	Galactofuranose	nd	3 ± 0.5	nd	nd
	Galactose	nd	nd	12 ± 2	7 ± 1
	Glucose	nd	nd	nd	22 ± 3
	Lactose	nd	7 ± 1	165 ± 15	57 ± 7
	Maltose	nd	nd	3 ± 0.4	3 ± 0.4
	Mannose	9 ±1	nd	nd	6 ± 1
	Myo-Inositol	nd	12 ± 5	130 ± 4	30 ± 3
	N-Acetyl-glucosamine	nd	nd	2 ± 0.5	nd
	Psicose	8 ± 1	nd	76 ± 17	19 ± 2
	Sucrose	nd	4 ± 1	506 ± 37	1097 ± 76
	Talose	nd	nd	21 ± 10	nd
	Threose	nd	nd	3 ± 0.4	nd
	Trehalose	nd	8 ± 3	9 ± 1	31 ± 4
	Turanose	42 ± 7	nd	nd	17 ± 2
Sugar acid	Arabinonic acid	nd	nd	2 ± 0.2	nd
	Galactaric acid	nd	nd	nd	2 ± 0.4
	Glyceric acid	7 ± 1	5 ± 1	nd	nd
	Ribonic acid	8 ± 1	8 ± 6	3 ± 1.5	5 ± 1
	Threonic acid	110 ± 13	8 ± 2	5 ± 3	3 ± 0.2
Sugar alcohol	Glucitol	7474 ± 886	nd	nd	nd
	Mannitol	nd	2 ± 0.1	nd	12 ± 2
	meso-Erythritol	453 ± 46	3 ± 0.1	nd	nd
	Scyllo-Inositol	153 ± 17	7 ± 1	2 ± 0.4	nd
	Sorbitol	10 ± 2	nd	nd	nd
Amino acid	2-Methylalanine	8 ± 1	nd	nd	nd
	Alanine	nd	2 ± 0.5	nd	nd
	Aspartic acid	24 ± 3	6 ± 2	nd	nd
	beta-Alanine	nd	nd	2 ± 0.2	nd
	Glutamic acid	109 ± 11	nd	nd	nd
	Glutamine	7 ± 1	nd	nd	nd
	Glycine	13 ± 1	6 ± 0.5	20 ± 1	7 ± 0.4
	Leucine	8 ± 1	nd	nd	nd
	Lysine	6 ± 1	nd	nd	nd
	Phenylalanine	10 ± 1	nd	nd	nd
	Proline	110 ± 19	nd	3 ± 1.5	nd
	Serine	8 ± 1	nd	nd	nd
	Threonine	7 ± 1	2 ± 0.3	nd	nd
	Valine	19 ± 2	nd	nd	nd
Fatty acid	9-Octadecenoic acid	nd	1 ± 0.1	nd	nd
	Butanoic acid	147 ± 14	nd	2 ± 0.2	nd
	Myristic acid	nd	3 ± 1	nd	nd
	Palmitic Acid	51 ± 6	18 ± 6	6 ± 1	nd
	Pentanoic acid	nd	3 ± 1	3 ± 0.2	nd
	Stearic acid	5 ± 2	6 ± 2	1 ± 0.1	nd

Fatty nitrile	Oleanitrile	nd	2 ± 0.4	nd	nd
Organic acid	Citric acid	7 ± 1	4 ± 2	nd	nd
	Lactic Acid	nd	261 ± 87	29 ± 5	38 ± 3
Organic compound	Bis(2-ethylhexyl) phthalate	nd	388 ± 69	351 ± 45	395 ± 6
	1,3,5-Benzetriol	17 ± 1	nd	nd	nd
	1,3-Propanediol	nd	5 ± 1		4 ± 0.2
	Ethanolamine	6 ± 1	19 ± 6	15 ± 2	7 ± 0.2
	Oxalic acid	nd	15 ± 9	6 ± 1	6 ± 1
	Succinic anhydride	nd	nd	4 ± 2	nd
Carboxylic acid	Cyclohexaneacetic acid	nd	2 ± 0.6	nd	nd
	Butanedioic acid	nd	14 ± 3	3 ± 0.4	nd
	Gluconic acid	nd	4 ± 2	nd	3 ± 0.5
	Malic acid	5 ± 1	nd	nd	nd
	Propanedioic acid	nd	2 ± 1	nd	nd
	Tartronic acid	nd	nd	nd	2 ± 0.3
Uronic acid	D-Glucuronic acid	45 ± 6	nd	nd	nd
Polyol comp.	Glycerol	nd	81 ± 18	57 ± 10	51 ± 4
	Pinitol	nd	nd	4 ± 1	nd
Diterpenoids	Dehydroabietic acid	nd	4 ± 1	nd	nd

Metabolite content is shown as µg g^{-1} DW of seaweed following latest rule of expressing data (http://web.ics.purdue.edu/~lewicki/physics218/significant)

DW: Dry Weight; ‘nd’: not detected

IS: *Iyengaria stellata*; SA: *Spatoglossum asperum*; SP: *Stoechospermum polypodioides*; SL: *Sargassum longifolium*

Table S3: Correlation matrix plots for PCA analysis of different activities (total antioxidant, scavenging, reducing and anti-proliferative) and contents (TPC and TFC) of the selected brown seaweeds.

Correlation matrix (Pearson):

Variables	Antioxidant	Scavenging	Reducing	TPC	TFC	AP-HeLa	AP-Huh
Antioxidant	**1**	0.629	0.401	-0.682	0.163	-0.397	0.924
Scavenging	0.629	**1**	0.930	**-0.989**	0.320	-0.822	0.299
Reducing	0.401	0.930	**1**	-0.937	0.042	-0.646	0.019
TPC	-0.682	**-0.989**	-0.937	**1**	-0.195	0.733	-0.354
TFC	0.163	0.320	0.042	-0.195	**1**	-0.789	0.169
AP-HeLa	-0.397	-0.822	-0.646	0.733	-0.789	**1**	-0.170
AP-Huh	0.924	0.299	0.019	-0.354	0.169	-0.170	**1**

Values in bold are different with a significance level alpha=0.05

p-values (Pearson):

Variables	Antioxidant	Scavenging	Reducing	TPC	TFC	AP-HeLa	AP-Huh
Antioxidant	**0**	0.371	0.599	0.318	0.837	0.603	0.076
Scavenging	0.371	**0**	0.070	**0.011**	0.680	0.178	0.701
Reducing	0.599	0.070	**0**	0.063	0.958	0.354	0.981
TPC	0.318	**0.011**	0.063	**0**	0.805	0.267	0.646
TFC	0.837	0.680	0.958	0.805	**0**	0.211	0.831
AP-HeLa	0.603	0.178	0.354	0.267	0.211	**0**	0.830
AP-Huh	0.076	0.701	0.981	0.646	0.831	0.830	**0**

Coefficients of determination (Pearson):

Variables	Antioxidant	Scavenging	Reducing	TPC	TFC	AP-HeLa	AP-Huh
Antioxidant	**1**	0.395	0.161	0.466	0.027	0.158	0.853
Scavenging	0.395	**1**	0.865	0.978	0.102	0.675	0.089
Reducing	0.161	0.865	**1**	0.877	0.002	0.417	0.000
TPC	0.466	0.978	0.877	**1**	0.038	0.537	0.125
TFC	0.027	0.102	0.002	0.038	**1**	0.623	0.029
AP-HeLa	0.158	0.675	0.417	0.537	0.623	**1**	0.029
AP-Huh	0.853	0.089	0.000	0.125	0.029	0.029	**1**

AP: Anti-proliferative activity on HeLa/ Huh-7 cell lines

Scatter plots:

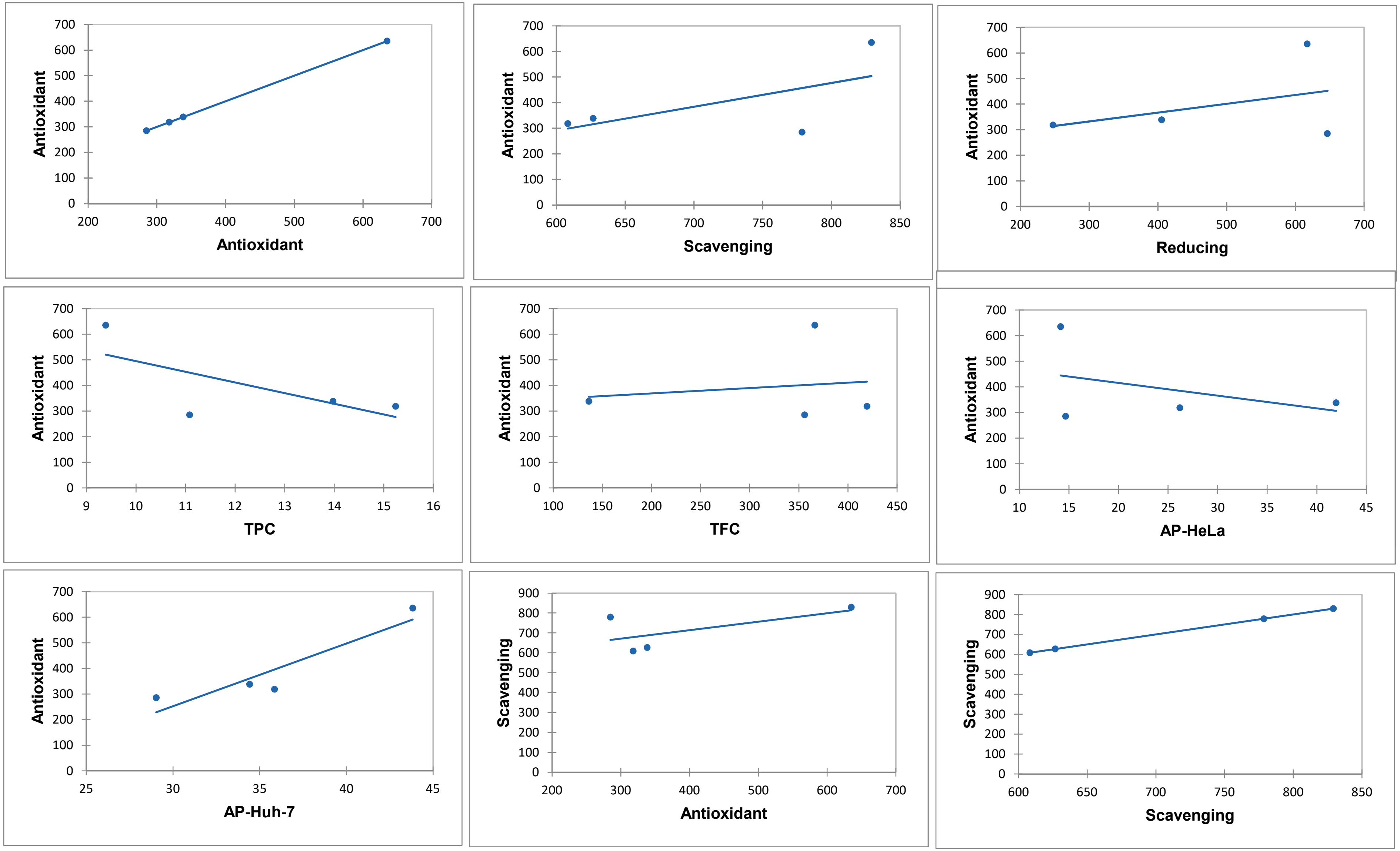

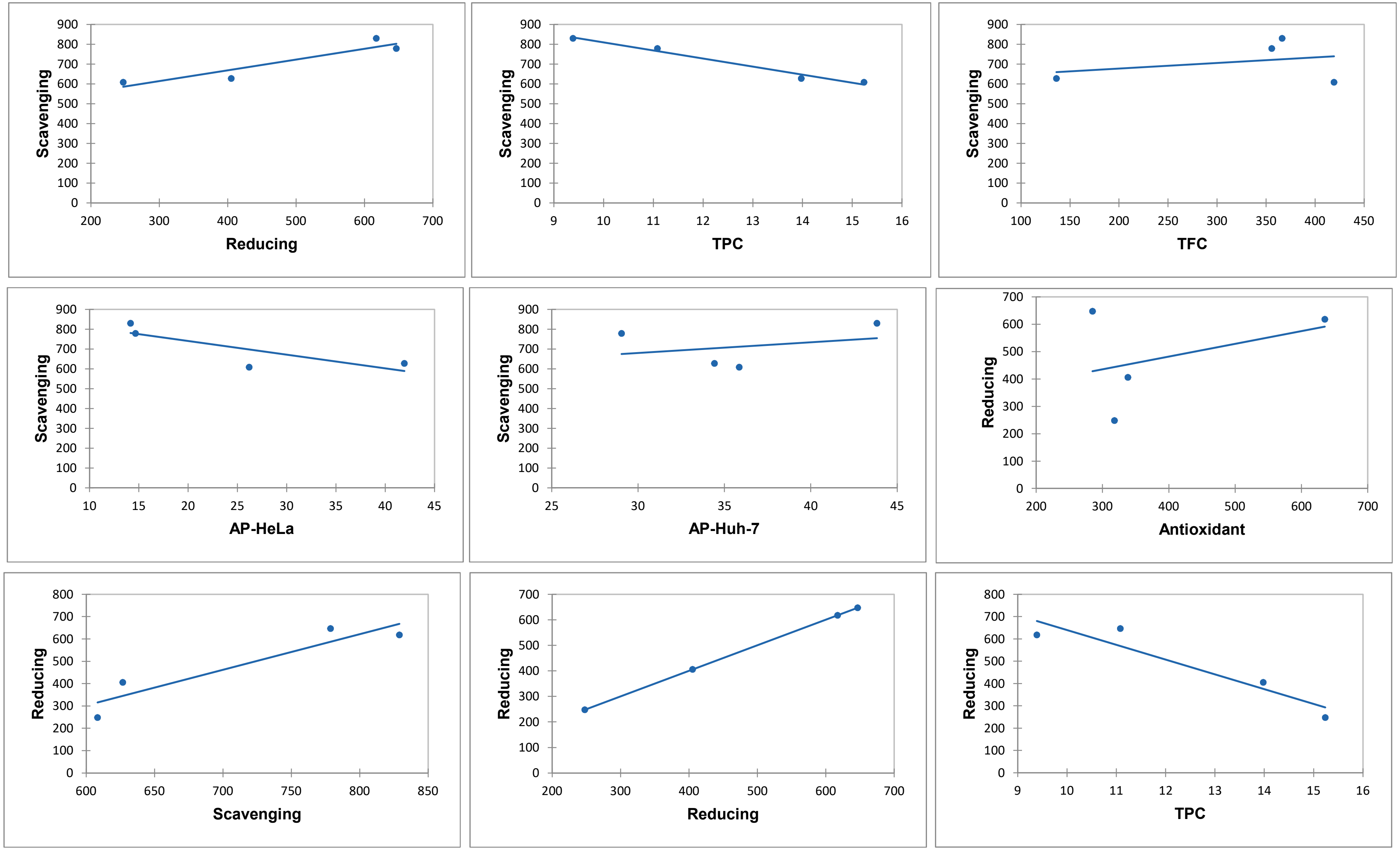
Scavenging
0
100
200
300
400
500
600
700
800
900
200
300
400
500
600
700
Reducing
Scavenging
0
100
200
300
400
500
600
700
800
900
9
10
11
12
13
14
15
16
TPC
Scavenging
0
100
200
300
400
500
600
700
800
900
100
150
200
250
300
350
400
450
TFC
Scavenging
0
100
200
300
400
500
600
700
800
900
10
15
20
25
30
35
40
45
AP-HeLa
Scavenging
0
100
200
300
400
500
600
700
800
900
25
30
35
40
45
AP-Huh-7
Reducing
0
100
200
300
400
500
600
700
200
300
400
500
600
700
Antioxidant
Reducing
0
100
200
300
400
500
600
700
800
600
650
700
750
800
850
Scavenging
Reducing
0
100
200
300
400
500
600
700
200
300
400
500
600
700
Reducing
Reducing
0
100
200
300
400
500
600
700
800
9
10
11
12
13
14
15
16
TPC

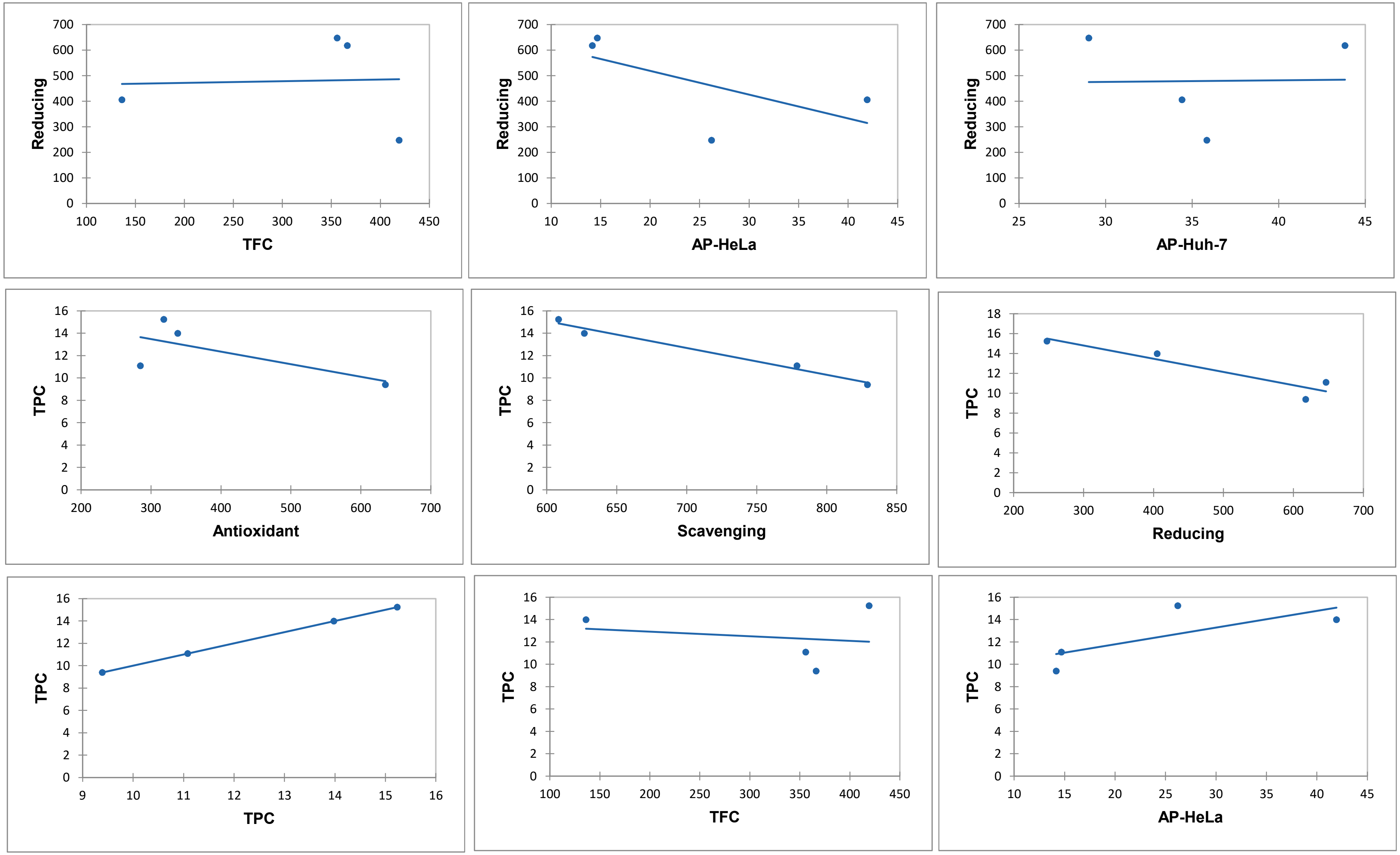
Reducing
TFC
Reducing
AP-HeLa
Reducing
AP-Huh-7
TPC
Antioxidant
TPC
Scavenging
TPC
Reducing
TPC
TPC
TPC
TFC
TPC
AP-HeLa

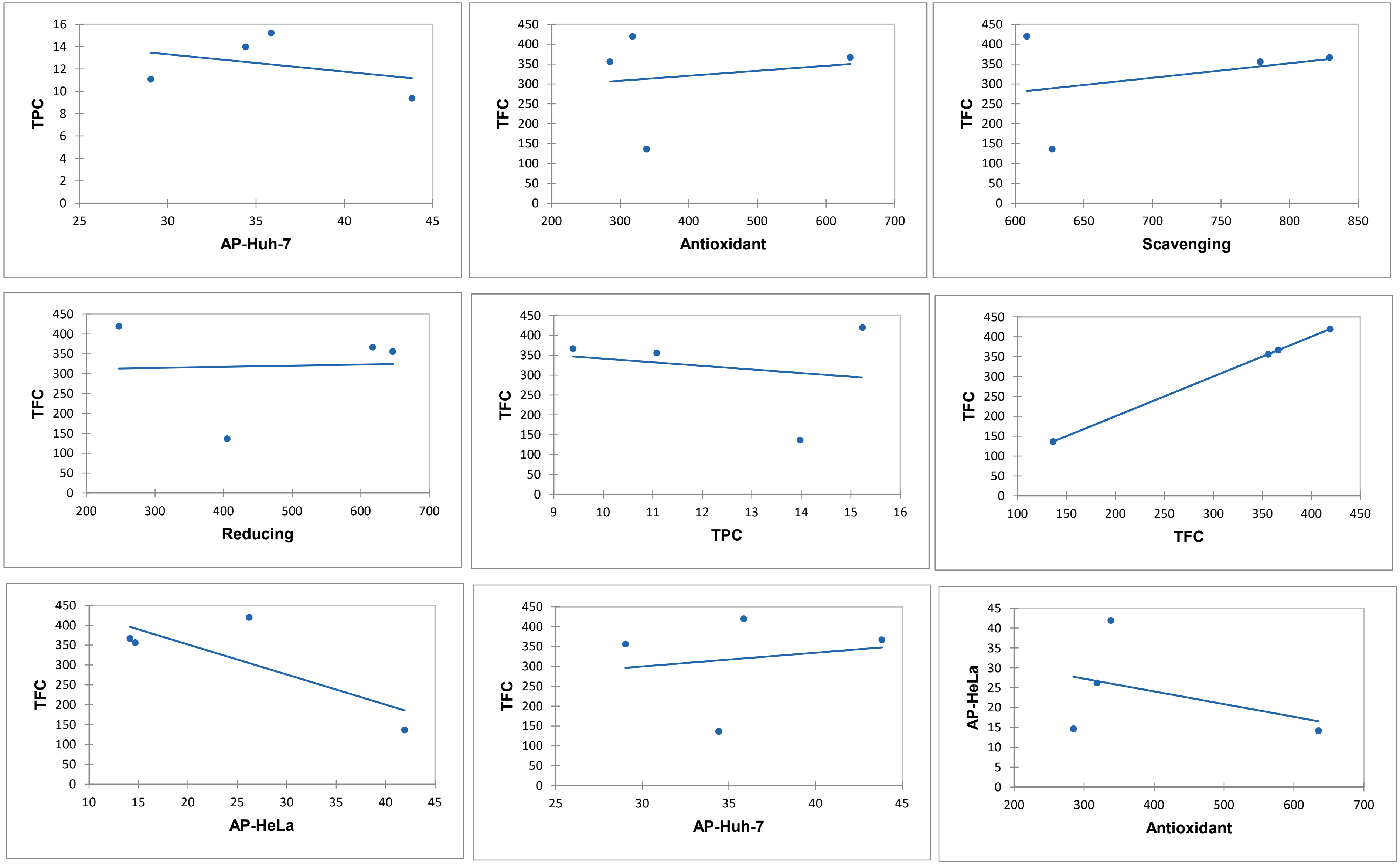
TPC
AP-Huh-7
TFC
Antioxidant
TFC
Scavenging
TFC
Reducing
TFC
TPC
TFC
TFC
TFC
AP-HeLa
TFC
AP-Huh-7
AP-HeLa
Antioxidant

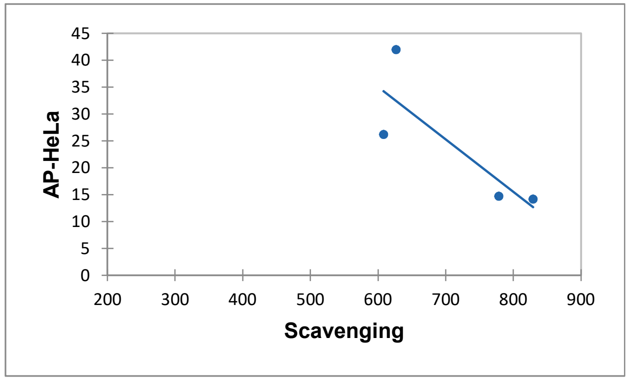
AP-HeLa
0 5 10 15 20 25 30 35 40 45
200 300 400 500 600 700 800 900
Scavenging

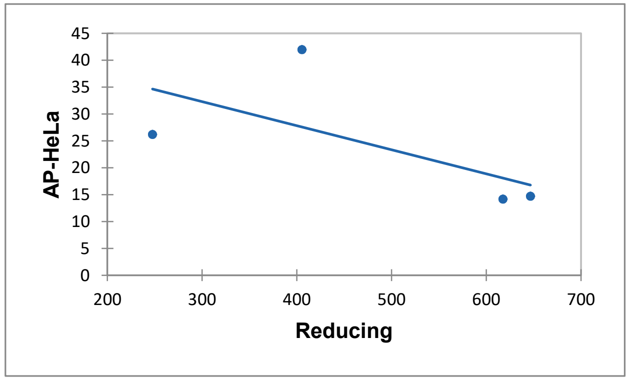
AP-HeLa
0 5 10 15 20 25 30 35 40 45
200 300 400 500 600 700
Reducing

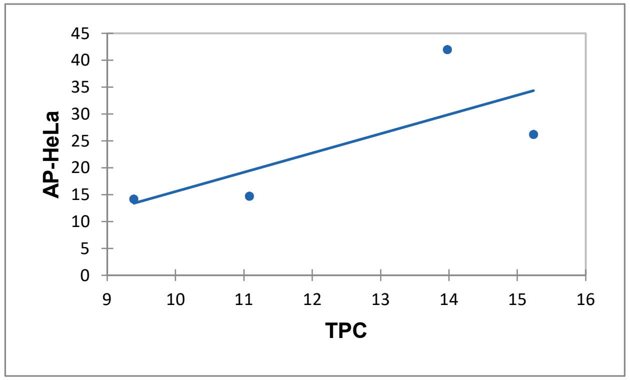
AP-HeLa
0 5 10 15 20 25 30 35 40 45
9 10 11 12 13 14 15 16
TPC

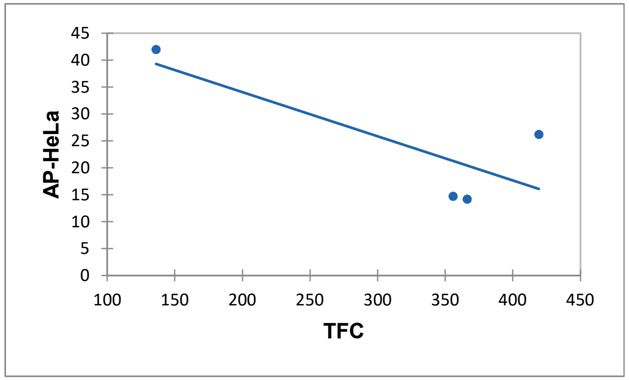
AP-HeLa
0 5 10 15 20 25 30 35 40 45
100 150 200 250 300 350 400 450
TFC

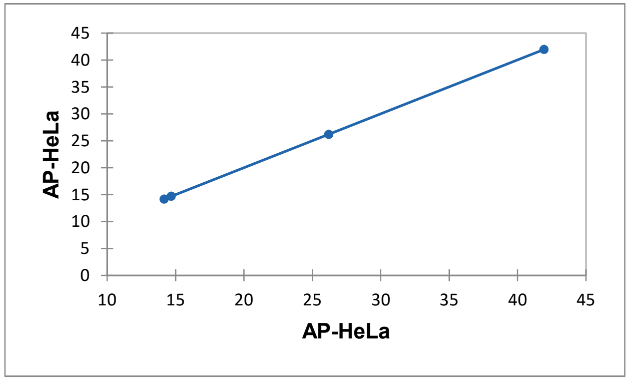
AP-HeLa
0 5 10 15 20 25 30 35 40 45
10 15 20 25 30 35 40 45
AP-HeLa

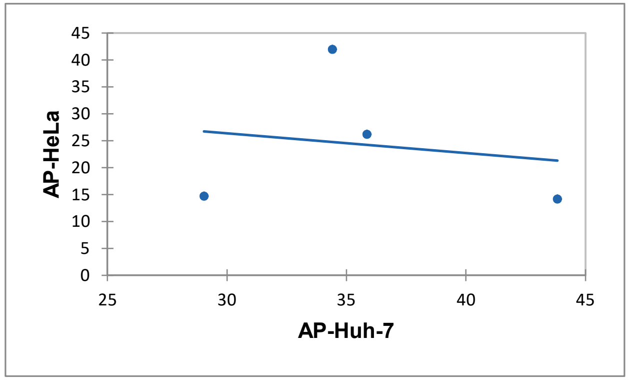
AP-HeLa
0 5 10 15 20 25 30 35 40 45
25 30 35 40 45
AP-Huh-7

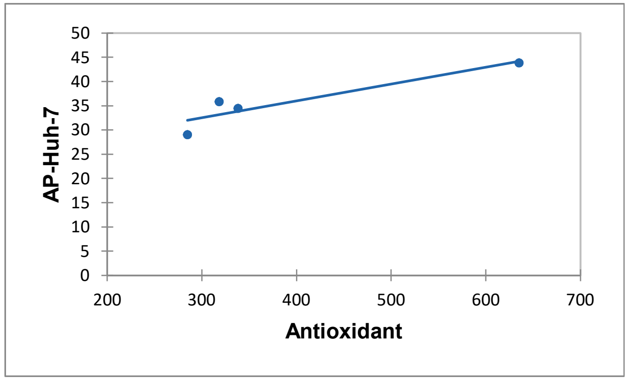
AP-Huh-7
0 5 10 15 20 25 30 35 40 45 50
200 300 400 500 600 700
Antioxidant

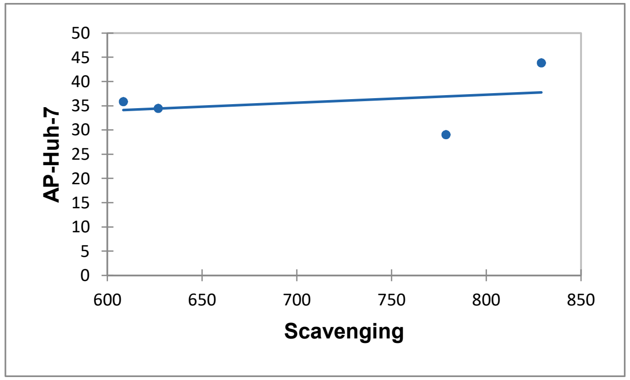
AP-Huh-7
0 5 10 15 20 25 30 35 40 45 50
600 650 700 750 800 850
Scavenging

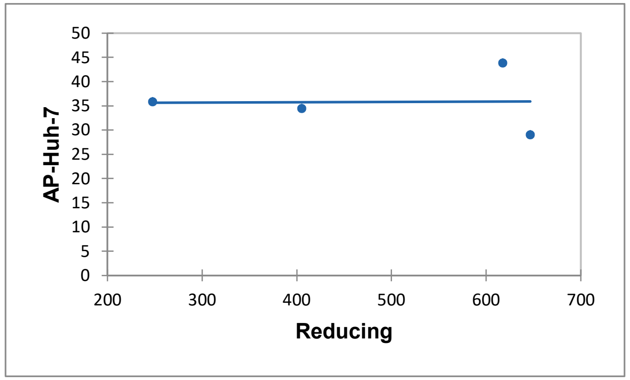
AP-Huh-7
0 5 10 15 20 25 30 35 40 45 50
200 300 400 500 600 700
Reducing

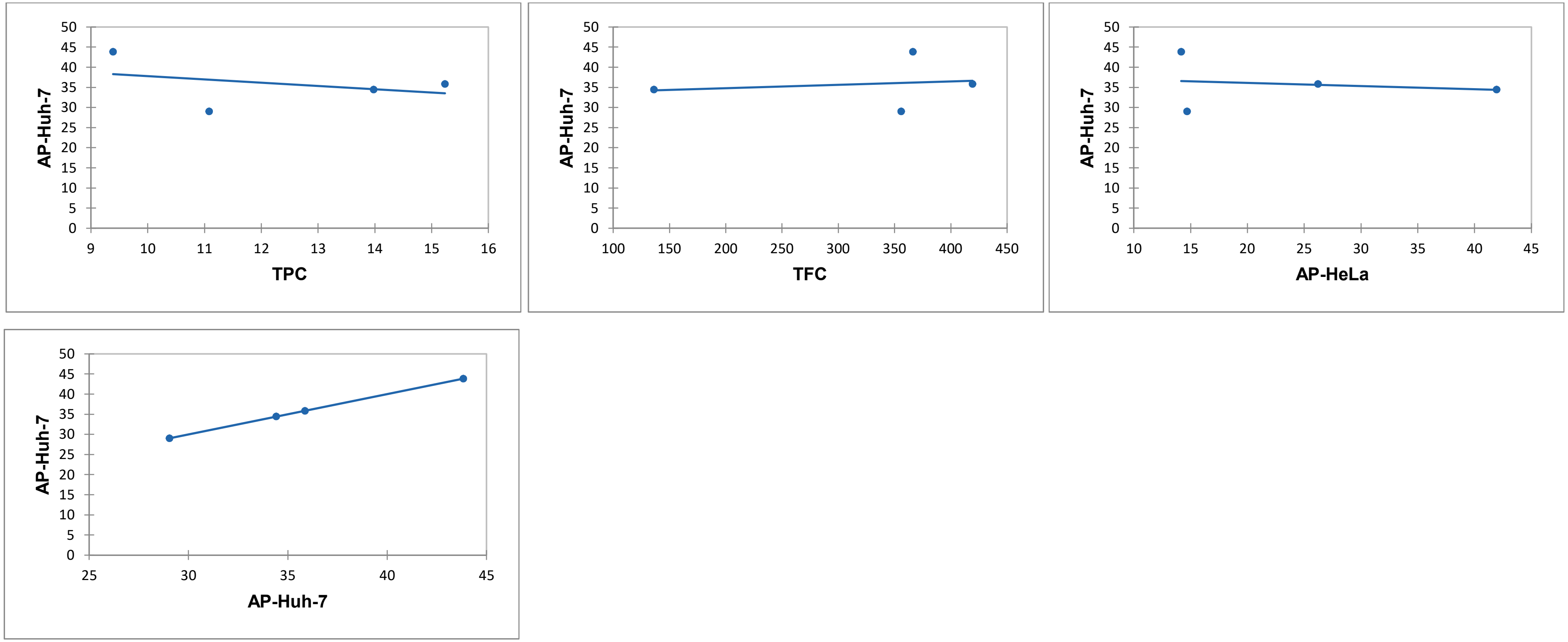
AP-Huh-7
TPC
AP-Huh-7
TFC
AP-Huh-7
AP-HeLa
AP-Huh-7
AP-Huh-7
